# British Medicinal Plants

**Published:** 1890-06

**Authors:** Alfred Heath

**Affiliations:** London, England


### BRITISH MEDICINAL PLANTS.

ALFRED HEATH, M. D., F. L. S., LONDON, ENGLAND.

The plants that I propose to mention in the following short sketches are such as are in common use in homœopathic practice or occur in homœopathic literature. My purpose in writing them is to show that medicines that were used formerly in an empirical manner as infallible remedies for the cure of disease, are now ascertained by the homœopathic "provings" on healthy people to be capable of *producing* the same conditions or symptoms that they were said to cure, thereby *proving* the truth of the homœopathic law of cure, *Similia similibus curantur*.

Further, by giving the common as well as the botanical names, I trust to interest the readers of THE HOMŒOPATHIC PHYSICIAN, by describing some of the virtues of plants that are well-known garden favorites, or wild flowers; and, lastly, that useful and practical information may be obtained concerning their use in disease when far from medical help. When in the country and wanting certain medicines, I have often been glad to find in the garden or hedge-row what could not be obtained at a shop. I propose, also, to make some mention of well-known plants that are not truly British for two reasons: first, they are well-known and "proven" homœopathic remedies, and also, secondly, although aliens they are in most cases thoroughly acclimatized, and capable of reproducing themselves by seeding, and many of them are *included in works on English botany*. I propose to take them in their botanical order and consequently commence with:

### ORDER 1, RANUNCULACEÆ.

*Clematis Vitalba* (The Traveler's Joy).—This beautiful climber is known to all, its dark-green foliage climbing thickly over almost every hedge in chalky or limestone districts. It is one of the earliest signs of coming spring, its abundance of white, almond-scented flowers later on, add their beauty to the fields, and in the autumn the hedge-rows are white with its feathery seed-plumes. This plant, like most others of the order, is very acrid, its leaves producing a warmth on the tongue, and if chewed for a short time causing blisters. They also blister and ulcerate the skin when rubbed on it, although in France the young sprouts are boiled and eaten as a vegetable like its namesake, better known in homœopathic materia medica (*Clematis erecta*). It was used internally as a cure for the lues venerea, certain forms of scrofula, and rheumatism. It has not been regularly " proven," but it cures many conditions that the " proven " (*C. erecta*) has produced, and probably the two plants closely resemble each other in their medicinal effects.

*Clematis Erecta* (Upright Virgin's Bower).—This plant is an alien, but grows so freely in our gardens that I cannot pass it over, especially as it is " a proven " and a most important homœopathic remedy. The same virtues are attributed to this plant in times past, as to the preceding. According to the homœopathic proving, it produced, on healthy persons, swelling of testes and scrotum, with painful sensitiveness, violent pains in left spermatic cord (in women it has cured glandular indurations of breasts, painful to touch). Rheumatic-like pains (aching, drawing, tearing), in limbs, and a great number of symptoms, affecting all parts of the body.

*Anemone Pulsatilla* (the Pasque Flower).—This plant, although very much like the *Pulsatilla nigricans* of the homœopathic pharmacopœia, is not the same, and should never be used for it. It flowers in the early spring *only*, and where common, as on the Gog and Magog hills, the pastures, it is said, are tinged with its elegant purple flowers. It is, however, a rare plant in Britain. It is found on grassy pastures and chalky declivities in several parts of the country, especially Hertfordshire. Its flowers are lighter in color than *P. nigricans*, and are *upright*, whereas the *Pulsatilla nigricans* flowers *twice* in the year, and has beautiful, dark, almost black-blue flowers, much more hairy, white, soft, silky hairs, much smaller flowers and *reflexed*, with the petals bent back at the top. The medicinal virtues of *A. Pulsatilla* are similar to those of *P. nigricans*, and it has been used with considerable success in diseases of women—headaches depending on functional derangements, inflammation of the eyes and eyelids, and malignant ulcers. It is useful in discharges from ears, nose, eyes, etc. It is very acrid; not "proven."

*Anemone Nemorosa* (the *Ranunculus Albus* of the pharmacopœias. The Wood Anemone or Wind Flower.)—Found in abundance in damp groves and thickets in early spring; is about four to eight inches high, flowers white, or with a purplish tinge. The whole plant is acrid and poisonous. Sheep eat it, but it is apt to disorder them violently. Horses, cows, and swine refuse it. The acrid volatile principle is so corrosive that it has been used externally as a blister, instead of Cantharides. It was used empirically in times past to promote the menstrual flow, and for the cure of leprosy. The juice was snuffed up the nose to promote discharges, and the root was chewed to cause expectoration on account of its acrid properties. It was found useful in headaches, in soreness and inflammation of the eyes and lids. Culpepper says: "Being made into an ointment and the eyelids anointed with it, it helps inflammation of the eyes, *whereby it is palpable that every stronger draws its weaker like.*" According to the same writer, it opens the mouths of the veins. All the foregoing states and conditions may be found in the "provings" of *Anemone Pratensis* or *Pulsatilla nigricans*; and all the Anemones probably contain the same active principle, *Anemonin.* Possibly if this plant were proven it would be found quite as good as our officinal plant, *Pulsatilla nigricans*, which latter does not grow here.

*Pulsatilla Nigricans* (*Anemone Pratensis*) Meadow Anemone, Wind Flower, an alien, but is found in our gardens, and is really a lovely thing to grow. This plant was received into the Edinburgh pharmacopœia during the life of Hahnemann, upon the authority of Baron Stoerck, who recommended it as an effectual remedy for most of the chronic diseases affecting the eye, particularly amaurosis, cataract, and opacity of the cornea, he also found it of great service in the nodes and nocturnal pains of syphilis, and in ulcers, caries, indurated glands, suppressed menses, herpetic eruptions, melancholy, and palsy. When chewed it inflames the tongue and fauces.

It produces, on the healthy, various kinds of inflammation of the eyes and eyelids, with pains and swelling. Lachrymation in the open air, dimness of sight, obscuration of sight, and a great many other eye symptoms; powerful and varied action on the sexual and urinary organs, eruptions on various parts of the body, which burn, itch, and bleed. Uterine spasms, suppression of the menses, great melancholy and depression of spirits, sadness, silent mood, weeping, and a great variety of motor disturbances, too numerous to mention; painful lameness, debility, rigid immobility of body, tingling, and sensation as if limbs had gone to sleep, tremulous weakness, trembling of the hands, and of limbs generally.

